# Investigation of Cytotoxicity of Biosynthesized Colloidal Nanosilver against Local *Leishmania tropica*: In Vitro Study

**DOI:** 10.3390/ma15144880

**Published:** 2022-07-13

**Authors:** Raghad Zein, Ibrahim Alghoraibi, Chadi Soukkarieh, Abdalrahim Alahmad

**Affiliations:** 1 Physics Department, Faculty of Sciences, Damascus University, Damascus P.O. Box 30621, Syria; ibrahim.alghoraibi@gmail.com; 2 Department of Basic and Supporting Sciences, Faculty of Pharmacy, Arab International University, Daraa P.O. Box 30621, Syria; 3 Department of Animal Biology, Faculty of Sciences, Damascus University, Damascus P.O. Box 30621, Syria; soukkarieh@gmail.com; 4 Institute of Technical Chemistry, Leibniz University Hannover, Callinstrasse 5, 30167 Hannover, Germany

**Keywords:** nanosilver, colloidal, *Eucalyptus camaldulensis*, SEM, NTA, zeta potential, *Leishmania tropica*

## Abstract

Leishmaniasis is one of the biggest health problems in the world. Traditional therapeutic methods still depend on a small range of products, mostly chemically. However, the treatment with these drugs is expensive and can cause serious adverse effects, and they have inconsistent effectiveness due to the resistance of parasites to these drugs. The treatment of leishmanial disease has always been a challenge for researchers. The development of nanoscale metals such as silver has attracted significant attention in the field of medicine. The unique characteristic features of silver nanoparticles (AgNPs) make them effective antileishmanial agents. In recent years, green nanotechnology has provided the development of green nanoparticle-based treatment methods for Leishmaniasis. Although there are many studies based on green nanoparticles against Leishmania parasites, this is the first study on the antileishmanial effect of biosynthesized AgNPs using an aqueous extract of *Eucalyptus camaldulensis* leaves (AEECL) as a reducing agent of silver ions. Different parameters such as AgNO_3_ concentration, AEECL concentration, and reaction time were studied to investigate the optimum factors for the preparation of stable and small-sized silver nanoparticles. The spherical shape of colloidal nanosilver (CN-Ag) was confirmed by atomic force microscope (AFM) and scanning electron microscope (SEM) images with sizes of 27 and 12 nm, respectively. A high density of nanoparticles with a small size of 10 nm has been confirmed from dynamic light scattering (DLS) analysis. The zeta potential value of 23 mV indicated that colloidal silver nanoparticles were stable. The nano-tracker analysis (NTA) showed the Brownian motion of silver nanoparticles with a hydrodynamic diameter of 31 nm. The antioxidant property of CN-Ag was determined using the stable radical 2,2-Diphenyl-1-picrylhydrazyl (DPPH) assay. In this study, a significant cytotoxic effect of biosynthesized CN-Ag has been shown against *Leishmania tropica* parasites at low concentrations (1.25, 2.5, and 3.75 µg/mL). These results could be used as a future alternative drug or could be a supportive treatment for Leishmaniasis.

## 1. Introduction

Leishmaniasis is a parasitic infection caused by different species of Leishmania protozoa. Leishmaniasis is considered endemic in 98 countries on 4 continents. It is estimated that approximately 1 million new cases of cutaneous leishmaniasis and 90,000 cases of visceral leishmaniasis occur worldwide each year according to the World Health Organization (WHO) [1], and the most prevalent cases were in the Middle East, especially in Syria with more than 40,000 cases a year [2]. Early case detection and treatment are the most important control measures for leishmaniasis. Therapeutic methods still depend on a small range of products, mostly chemically, and contain compounds of pentavalent antimony (pentostam and glucantime). These drugs can present serious side effects, such as pancreatitis, hepatotoxicity [3], and cardiotoxicity, and sometimes, they have inconsistent effectiveness due to the microorganisms’ drug resistance [4,5]. The treatment of leishmaniasis disease has always been a challenge for researchers. Nanomedicine is one of the promising fields that has been continuously growing and keeping up hope to develop new promising alternatives that can cure leishmaniasis [6,7]. The development of nanoscale metals such as silver, gold, zinc, and titanium has attracted significant attention in the field of medicine. Silver nanoparticles occupy a prominent place in the series of such metals due to their antimicrobial [8,9,10], anticancer, and antileishmanial properties, which allowed them to be applicable in a wide variety of therapeutic uses [11,12,13,14]. The unique characteristic features of silver nanoparticles are related to their small size and large surface area. Additionally, the high ability of silver nanoparticles to generate reactive oxygen species makes them an effective material against Leishmania [15,16]. Lately, most researchers have focused on alternative methods for the synthesis of silver nanoparticles that do not necessitate the use of hazardous chemical compounds in the production procedure, such as biochemistry and green chemistry [17]. These methods mainly rely on biomolecules from natural sources [18] such as yeasts [19], fungi [20], bacteria [21], and plant extracts [22,23,24,25]. These biomolecules have been used as reducing and stabilizing agents for converting silver ions to silver nanoparticles. Green synthesized AgNPs showed an effective role against Leishmania parasites according to previous literature [26,27]. Interestingly, they were found to be more effective than chemically prepared silver nanoparticles, as reported in Ullah et al. [28]. Hashemi et al. revealed that green synthesized Ag nanoparticles can inhibit the growth of parasite cell lines more effectively than glucantime [29]. In this work, CN-Ag has been synthesized using an aqueous extract of *Eucalyptus camaldulensis* leaves, which was considered as a prolific source for several biologically active metabolites. The leaves’ extract of this plant showed considerable antimicrobial [30], antifungal [31], and antioxidant activity [32]. There are few articles published on the antileishmanial effect of different *Eucalyptus camaldulensis* extracts as alcoholic, methanolic, and aqueous extracts [33,34]. The alcoholic and methanolic extracts were more effective than the aqueous. These studies revealed that the aqueous extract of *Eucalyptus camaldulensis* leaves has a significant effect against Leishmania parasites at concentrations of more than 1000 µg/mL. To the best of our knowledge, this is the first in vitro study showing the potent antileishmanial effect of colloidal nanosilver biosynthesized using aqueous extract of *Eucalyptus camaldulensis* against *Leishmania tropica*.

## 2. Materials and Methods

### 2.1. Materials

Silver nitrate (AgNO_3_) and all other chemicals were purchased from Sigma-Aldrich. Freshly prepared distilled water was used throughout the experiment.

### 2.2. Synthesis of Silver Nanoparticles

The preparation method of silver nanoparticles has been mentioned in our previous work in detail [35]. Briefly, an aqueous extract of dried *Eucalyptus camaldulensis* leaves has been prepared. The extract has been used as a reducing and capping agent in the synthesis process of silver nanoparticles. A specific amount of AEECL was added to the silver nitrate (AgNO_3_) solution at room temperature. The color of the mixture changing from yellow to dark brown indicates the formation of silver nanoparticles. The reaction mixture was kept in the dark at room temperature of 25 °C for 24 h to be sure that the reaction was completed. After that, nanoparticles were separated by centrifugation at 10,411× *g* for 5 min, the residue was collected and washed four times with deionized water (DI), and then with ethanol by centrifugation to get rid of silver ions and any uncoordinated biological materials. The colloidal sample was prepared by adding a small amount of DI water to the washed silver nanoparticles and dissolving them using the ultrasonic bath for 15 min. Different parameters such as AgNO_3_ concentration, AEECL concentration, and reaction time were studied to investigate the optimum factors, which led to the preparation of stable and small-sized silver nanoparticles.

### 2.3. Characterization of Silver Nanoparticles

The resulting silver nanoparticles were examined using UV-Vis spectrophotometer, dynamic light scattering (DLS), scanning electron microscope (SEM), energy dispersive X-ray spectrum (EDX), atomic force microscope (AFM), and nano-tracker analysis (NTA) techniques. The preliminary characterization of CN-Ag was carried out using a UV-visible spectrophotometer (Cary 5000) and a cell with a 1 cm path length. The analysis was performed after diluting a small aliquot of the suspension into deionized water (1:4). The baseline was corrected using the prepared AEECL. The particle size distribution in solution and the zeta potential of the prepared CN-Ag were determined by the DLS technique using the Zetasizer Nano ZS from Malvern, Worcestershire, UK. All measurements were recorded in triplicate. The surface morphology (size and shape) of silver nanoparticles was characterized by high-resolution scanning electron microscopy (HRSEM) (JEOL GmbH JSM-6700F SEM, Tokyo, Japan). The sample was prepared by drop-casting, where a small drop of diluted CN-Ag was placed on a carbon-coated graphite grid and allowed to dry by using a hairdryer for evaporating the solvent. Then, readings were taken at ×150 K magnification with steady voltage (20 kV). EDX analysis was performed to confirm the presence of elemental silver. Particle size was also examined using AFM (Nanosurf easyScan2, Listal, Switzerland), using tapping mode in the air at room temperature. The sample was prepared by drop-casting, where 100 µL of CN-Ag was placed on a glass substrate and allowed to dry at room temperature. The particle size distribution of CN-Ag was analyzed using the NTA technique (NanoSight LM10 system (NanoSight Ltd., Amesbury, UK)). Each sample was measured three times with a highly sensitive camera. 

### 2.4. Radical Scavenging Activity (DPPH)

The method was performed according to Li et al. [36]. The free radical scavenging activity of CN-Ag and standard ascorbic acid was determined using the stable radical DPPH: 100 μL of different concentrations (0.5, 1.25, 2.5, 5, 12.5 µg/mL) of CN-Ag was mixed with 3.9 mL of freshly prepared DPPH solution (0.1 mM in ethanol). Then, the mixture was incubated at room temperature in the dark for 30 min. The absorbance was recorded at 517 nm using a UV-Vis spectrophotometer. DPPH was used as a control and ethanol was used as a blank solution. The percentage of DPPH radical scavenging activity was determined using the following formula:

$$\mathrm{scavenging}\;\left(\%\right)=\frac{A_{c}-A_{S}}{A_{c}}$$

where A_c_ is the absorbance of the control and A_s_ is the absorbance of the sample.

### 2.5. Antileishmanial Activity

#### 2.5.1. Culture of Leishmania Parasite

Leishmania strains (*L. tropica*) used in this study were isolated from the patients and typed by PCR at the dermatology hospital of Damascus University, Syria. *Leishmania tropica* promastigotes were cultured at 26 °C in culture flasks containing RPMI-1640 supplemented with 10% heat-inactivated fetal bovine serum (FBS) (Sigma-Aldrich), 0.5 mm of L-glutamine (Sigma-Aldrich), and penicillin/streptomycin 100 U/mL. The growth of promastigotes was monitored daily using an inverted microscope (Optika 400×). Samples of promastigotes were fixed in formaline (1%) and counted using a hemocytometer with a 400× objective under standard light microscopy.

#### 2.5.2. Cytotoxicity Assay on *L. tropica* Promastigotes

Parasites were seeded as 1 × 106 promastigotes per well in 24-well flat-bottom microplates. The parasites were exposed to different concentrations of silver nanoparticles (0.125, 0.25, 0.5, 1.25, 2.5, and 3.75 µg/mL). The concentrations were prepared using an initial solution with a concentration of 25 µg/mL. The volume of parasites and nanoparticles in a well was completed with RPMI-1640 medium to 200 µL. In a negative control group, promastigotes were left without added nanoparticles, while in the positive control group, glucantime 30 μg/mL was added with the promastigotes. The plate was incubated for 24 and 48 h at 27 °C. After incubation, the proliferation of promastigotes of Leishmania was investigated by counting the number of parasites using a Neubauer chamber. The viability of parasites was calculated using the following equation:

$$\mathrm{viability}\left(\%\right)=\frac{\;\mathrm{number}\;\mathrm{of}\;\mathrm{treated}\;\mathrm{parasites}}{\mathrm{number}\;\mathrm{of}\;\mathrm{control}\;\mathrm{parasites}}*100$$

#### 2.5.3. Data Processing and Statistics

All experiments were repeated three times in triplicate wells. The results were expressed as the mean plus or minus the standard deviation. The analysis of variance (one-way ANOVA) of Origin Program version 8.5 was used to evaluate the results for significant differences. Values of *p* > 0.001 were considered statistically significant.

## 3. Results and Discussion

### 3.1. UV-Vis Spectroscopy Analysis

In this work, different parameters have been studied to achieve the maximum yield of nanoparticles of a certain shape and size, including the concentration of silver nitrate, the volume ratio of AEECL and silver nitrate, and incubation time. UV-Vis spectroscopy analysis provides a preliminary characterization of synthesized silver nanoparticles. The surface plasmon resonance peak could yield information about the size, shape, and aggregation status of nanoparticles.

#### 3.1.1. Effect of AgNO_3_ Concentration

**Figure 1:**
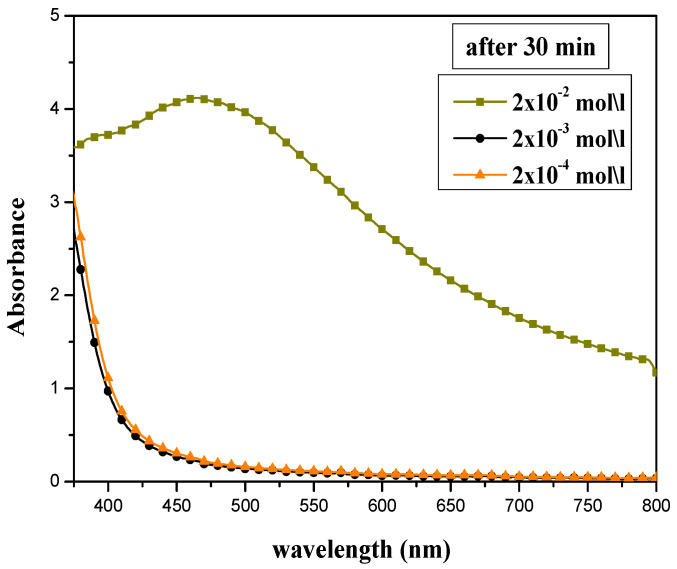
UV-Vis absorption spectra for silver nanoparticles synthesized at various concentrations of AgNO_3_: 2 × 10^−2^, 2 × 10^−3^, and 2 × 10^−4^ mol/L.

The absorption peak intensity of prepared silver nanoparticles was highly affected by the concentration of silver nitrate solution, as shown in Figure 1. Different AgNO_3_ concentrations (2 × 10^−2^, 2 × 10^−3^, and 2 × 10^−4^ mol/L) have been tested, and the volume ratio of AEECL and silver nitrate was kept constant at 10:10. The SPR peak appeared only at a higher concentration of silver nitrate of 2 × 10^−2^ mol/L. This may be due to an inadequate number of silver ions for bio-reduction in the samples at lower concentrations (2 × 10^−2^ and 2 × 10^−3^ mol/L), and despite the changing color, no absorption peak was observed. The concentration of silver nitrate of 2 × 10^−2^ mol/L was adopted in the next experiments.

#### 3.1.2. Effect of Volume Ratio of AEECL and Silver Nitrate

**Figure 2:**
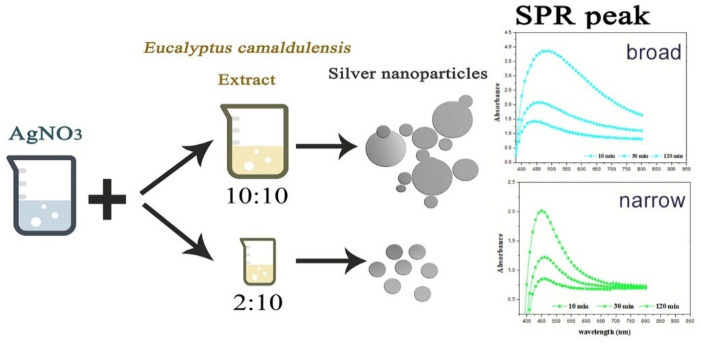
Scheme of the effect of AEECL concentration on the size of silver nanoparticles and SPR peak.

The effect of *Eucalyptus camaldulensis* leaf extract was studied using different volume ratios of AEECL to 0.02 M of silver nitrate solution (2:10, 5:10, 10:10). As the volume of AEECL increased, the quantity of reductants increased, and thus the rapid reduction rate of Ag^+^ to Ag^0^ led to the agglomeration phenomenon between particles, which was accompanied by a broad SPR peak (as shown in Figure 2).

**Figure 3:**
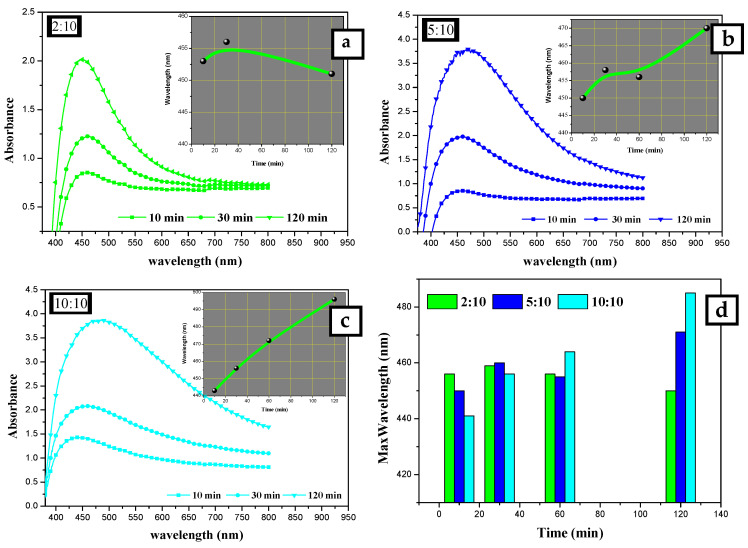
UV-Vis absorption spectra for silver nanoparticles synthesized at different extract to AgNO_3_ ratios: (**a**) 10:10, (**b**) 5:10, and (**c**) 2:10 mL:mL. (**d**) Changing λ_max_ of SPR peak at different reaction times: 10, 30, 120 min.

At a lower concentration of AEECL, a homogenous size of nanoparticles formed, which is consistent with a narrow SPR peak (see Figure 2). The SPR peaks in the UV-Vis spectra showed the best representation in a ratio of 2:10 with excellent enhancement in the absorption band intensity at 450 nm, as observed in Figure 3a. This result implies that the silver nanoparticles prepared at low volume ratios of AEECL and AgNO_3_ were very stable without agglomeration.

#### 3.1.3. Effect of Reaction Time

The other factor considered was the time. The absorption spectra were recorded for silver nanoparticles prepared using 10 mL of 2 × 10^−2^ mol/L of AgNO_3_ solution and different volumes of AEECL (2, 5, and 10 mL) at various time intervals (10, 30, and 120 min), as shown in Figure 3. Increasing the reaction time resulted in a gradual increase of SPR peak intensity, which indicates that more silver nanoparticles were formed as the period of the reaction time increased. The insets in Figure 3a–c illustrate the change of maximum wavelength with time (10, 30, and 120 min). At the high volume of AEECL to silver nitrate, a redshift in λ_max_ was indicated with an increasing reaction time. While at a low volume ratio of AEECL to AgNO_3_, the value of λ_max_ did not change drastically (see Figure 3d) and narrow-width SPR peaks have been observed, which indicate the homogeneous formation of silver nanoparticles with a small size.

To sum up, the suitable conditions for the synthesis of silver nanoparticles using an aqueous extract of dried *Eucalyptus camaldulensis* leaves were determined as: AgNO_3_ concentration was 2 × 10^−2^ mol/L and the volume ratio of AEECL extract to AgNO_3_ solution was 2:10.

**Figure 4:**
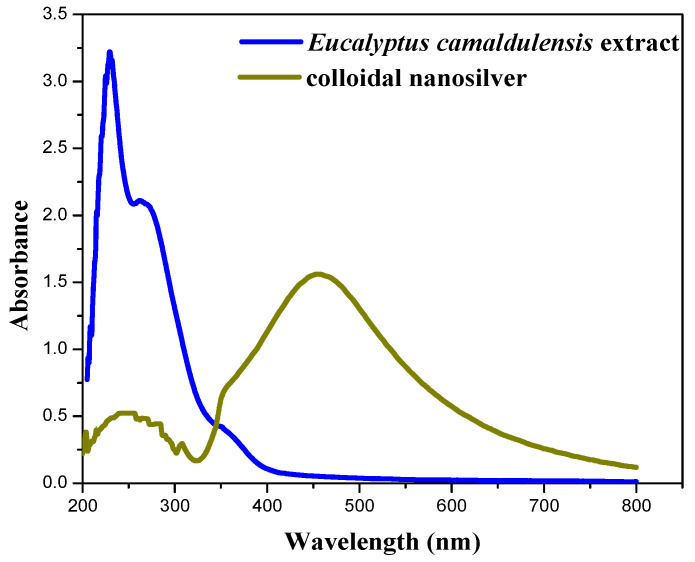
UV-Vis absorption spectra of aqueous extract of *Eucalyptus camaldulensis* leaves and colloidal nanosilver after centrifugation and washing processes (baseline was corrected using DI water).

Figure 4 shows the UV-Vis absorption spectra of aqueous extract of *Eucalyptus camaldulensis* leaves and colloidal nanosilver after centrifugation and further washing processes. The baseline was corrected using DI water. The pure extract showed two absorption peaks at 230 and 270 nm, which indicate the presence of polyphenols and other phytochemicals that are responsible for the reduction of silver ions to silver metal [37,38]. These peaks almost disappeared after washing processes, which confirmed the additional role of the extract as a capping agent. The peak at 450 nm is due to silver nanoparticles.

### 3.2. Dynamic Light Scattering Analysis

**Figure 5:**
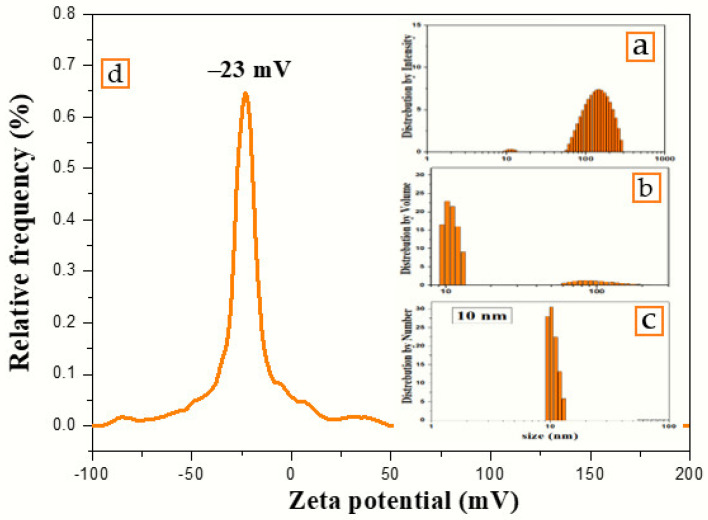
Particles’ size distribution by (**a**) intensity, (**b**) volume, (**c**) number, and (**d**) zeta potential of colloidal nanosilver synthesized using aqueous extract of *Eucalyptus camaldulensis* leaves.

The number, volume, and intensity particle size distributions of colloidal nanosilver synthesized using the volume ratio 2:10 of AEECL and AgNO_3_ solution are shown in Figure 5. The number-based distribution revealed narrow scattering centres with a mean diameter of 10 nm, while the intensity-based distribution observed a small number of nanoparticles with a large size around 100 nm. DLS results indicated 0.204 PDI, which depicts that the nanoparticles are well-dispersed in the used solvent, i.e., water. The high value of PDI is related to the different size of nanoparticles, as shown in the intensity and volume distribution. The zeta potential of the fabricated AgNPs determined in water as a dispersant was −23 mV, as indicated in Figure 5. A negative value suggests that the surface of the nanoparticles was negatively charged, which caused a strong electrostatic repulsion force between the particles, and thus an increase in the stability of the formulation without adding any capping agent. This is very important for use for therapeutic purposes.

### 3.3. Scanning Electron Microscopy and EDX Analysis

**Figure 6:**
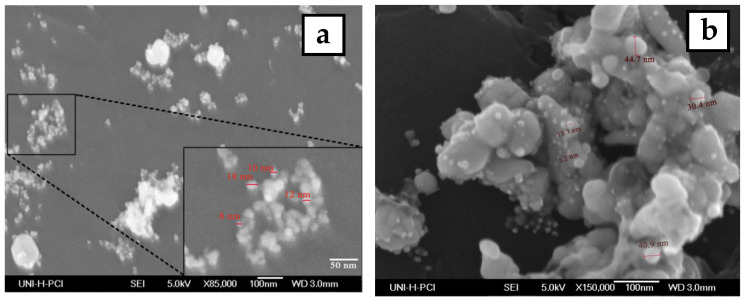

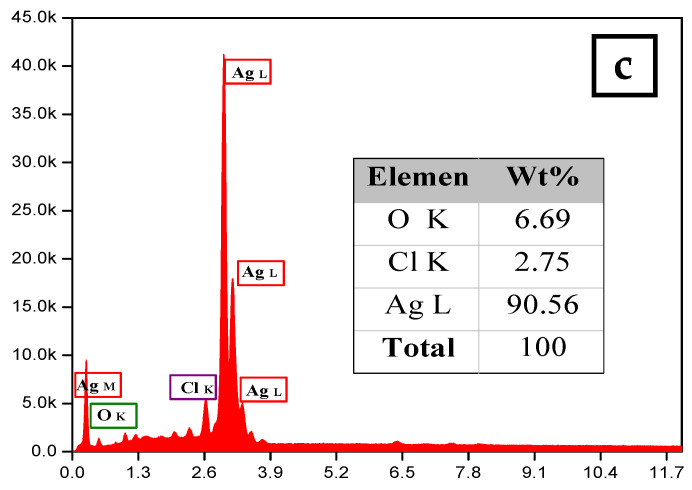
SEM images of silver nanoparticles prepared using different volume ratios of silver nitrate and ECL extract: (**a**) 2:10 and (**b**) 10:10. (**c**) EDX spectrum of silver nanoparticles.

The morphology and size of the synthesized silver nanoparticles for two-volume ratios of silver nitrate and ECL extract, 2:10 and 10:10, were determined by SEM images as shown in Figure 6a,b, respectively. The particles formed were spherical. The average size of nanoparticles was 12 and 27 nm at a low and high ratio of ECL extract, respectively. As the quantity of ECL extract increased, the nanoparticles became bigger, agglomerated, and had different sizes. This result corresponds to the redshift and wide width of the SPR peak in UV-Vis analysis at a ratio of 10:10. Additionally, the elemental composition of the silver NPs synthesized via AEECL was determined using the EDX detector shown in Figure 6. The intense sharp signal obtained at 3 KeV shows that silver was the main element (80.05 wt.%). The presence of other peaks of chlorine (7.18 wt.%) and oxygen (12.7 wt.%) may be ascribed to the presence of phytoconstituents of *Eucalyptus camaldulensis* leaves’ extract that adhered to the surface of nanoparticles.

### 3.4. Atomic Force Microscopy Analysis

**Figure 7:**
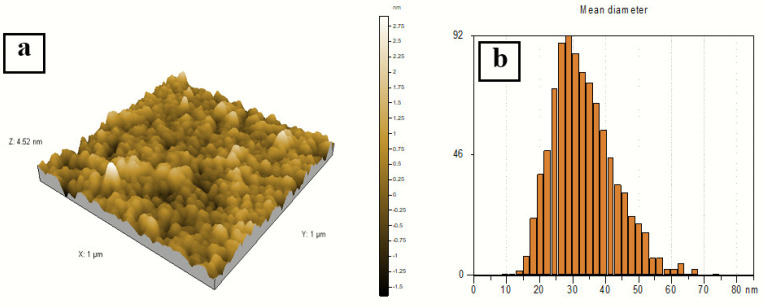
(**a**) 3D AFM image of silver nanoparticles. (**b**) Particle size distribution histogram of silver nanoparticles prepared at a volume ratio of silver nitrate and AEECL extract of 2:10.

A three-dimensional (3D) AFM image (1 × 1 µm) of the sample prepared at a 2:10 volume ratio of silver nitrate and AEECL is presented in Figure 7. Homogeneous and spherical shape and regular size distribution of silver nanoparticles with a mean size of 27 nm have been observed. The narrow distribution histogram of silver nanoparticles infers the uniform size and high homogeneity of Ag nanoparticles. The size of silver nanoparticles measured by AFM was bigger than that determined by DLS, which may be due to the method used to prepare the sample for AFM analysis.

### 3.5. NTA Analysis 

**Figure 8:**
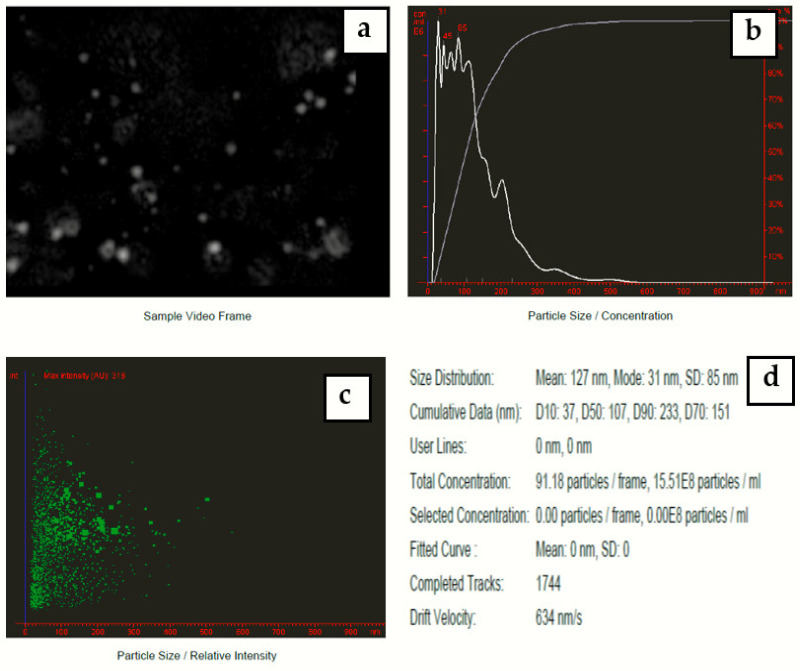
(**a**) Video frame, (**b**) 2D size vs. number particles’ distribution, (**c**) 2D size vs. intensity particles’ distribution, and (**d**) results report for colloidal nanosilver prepared at a volume ratio of 2:10 of silver nitrate and AEECL extract.

Nanoparticle tracking analysis measures the particle size by video tracking. The Brownian motion of each particle is followed simultaneously in real-time via video. An immediate idea of sample concentration and particle size is gained in seconds. Figure 8 shows a video frame and particle size distribution for AgNPs in solution. The mean size of silver nanoparticles-synthesized *Eucalyptus camaldulensis* leaves’ extract was 127 nm and mode size was 31 nm.

### 3.6. DPPH Assay for Antioxidant Activity

The antioxidant activity of synthesized CN-Ag was evaluated by the DPPH radical scavenging assay. Different aliquots were prepared as 0.5, 1.25, 2.5, 5, and 12.5 μg/mL. Ascorbic acid was used as a positive control (in the range of 1 to 5 μg/mL). The results obtained are summarized in Table 1. The scavenging ability of CN-Ag increased in a dose-dependent manner. The recorded scavenging ability for the lowest concentration of the synthesized CN-Ag (0.5 μg/mL) was 3.96 ± 0.53, and this scavenging ability was increased to 66.37 ± 0.91 when the concentration was increased to 12.5 μg/mL (average IC_50_ = 9.46 ± 0.59).

### 3.7. Anti-Promastigote Effect

**Figure 9:**
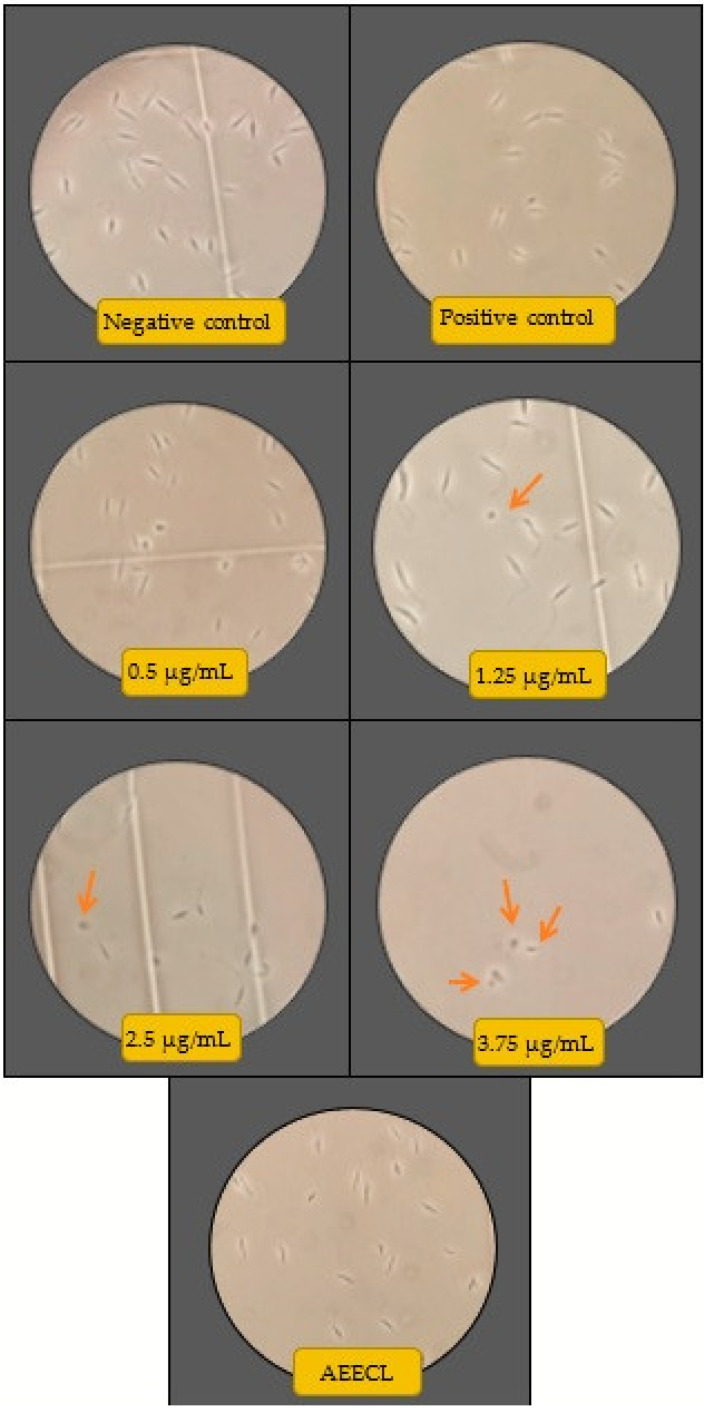
Microscopic views of parasites in the negative control (not exposed to silver nanoparticles), positive control (glucantime), AEECL (aqueous extract of *Eucalyptus camaldulensis* leaves 1000 µg/mL), and different concentrations of CN-Ag (0.5, 1.25, 2.5, and 3.75 µg/mL). Red arrows indicate the shrinkage and round shape of *Leishmania tropica* promastigotes.

**Figure 10:**
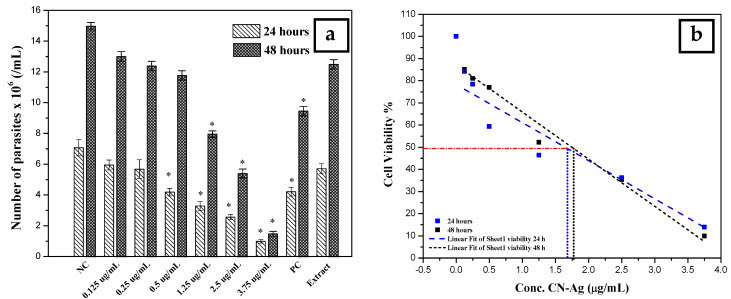
(**a**) Number of *L. tropica* parasites in the negative control (NC) (not exposed to silver nanoparticles), the positive control (PC) (exposed to glucantime), and the AEECL (aqueous extract of *Eucalyptus camaldulensis* leaves 1000 µg/mL), and at different concentrations of CN-Ag (0.125, 0.25, 0.5, 1.25, 2.5, and 3.75 µg/mL), after 24 and 48 h of incubation. Results are represented as mean ± SD of three independent experiments. Statistical significance was assessed by a one-way ANOVA: * *p* < 0.001 compared with the negative control. (**b**) Linear fit regression for curve of cell viability of *L. tropica* parasites at different concentrations of CN-Ag (0.125, 0.25, 0.5, 1.25, 2.5, and 3.75 µg/mL) after 24 and 48 h of incubation.

The antileishmanial activity of silver nanoparticles against *Leishmania tropica* promastigotes was determined for different concentrations (0.125, 0.25, 0.5, 1.25, 2.5, and 3.75 µg/mL) of colloidal nanosilver and compared with the negative control (not exposed to silver nanoparticles), positive control (exposed to glucantime), and AEECL (1000 µg/mL) after 24 and 48 h of incubation. The promastigotes in the negative control group have a spindle shape and a long flagellum, as seen in Figure 9. However, when the samples were exposed to CN-Ag, the shape and the number of *L. tropica* promastigotes changed. The concentration of 3.75 μg/mL was the most effective, strongly inhibiting the proliferation of *L. tropica* promastigotes, and a shrinkage round shape appeared (as indicated in Figure 9 by red arrows). The effect of different concentrations of CN-Ag on the number of *L. tropica* parasites after 24 and 48 h of incubation is shown in Figure 10a. A gradual decrease in the number of parasites appeared with increasing the concentrations of colloidal nanosilver. Statistically, significant differences were observed between the samples treated with CN-Ag and the negative control. This indicated the antileishmanial activity of CN-Ag against the tested promastigotes of leishmania. Figure 10b shows that each concentration of CN-Ag negatively affected the viability of *L. tropica* promastigotes. The percentage of viability after 24 h of exposure to CN-Ag (0.125, 0.25, 0.5, 1.25, 2.5, and 3.75 µg/mL) was 84%, 78%, 59%, 46%, 36%, and 14%, respectively. After 48 h, the results showed that the percentage of viability was 85%, 81%, 77%, 52%, 35%, and 10%, respectively. Besides, the percentage of viability for the glucantime group (positive control) was 60% and 62% after 24 and 48 h, respectively. In comparison with glucantime (30 µg/mL), it was obvious that CN-Ag with concentrations of 0.5, 1.25, 2.5, and 3.75 µg/mL was more effective. On the other hand, the aqueous extract of Eucalyptus (1000 µg/mL) inhibited the growth of promastigote by 20% and 18% after 24 and 48 h, respectively. This result confirms that the antileishmanial activity of colloidal nanosilver is due to silver nanoparticles, not to the phytochemicals on their surface.

The highest concentration of silver nanoparticles (3.75 µg/mL) showed the biggest suppressive effect on parasites, whereby it inhibited the growth of parasites by 90%. The IC_50_ was calculated using linear regression for a curve that obtained the effect of different concentrations of silver nanoparticles on *L. tropica* promastigotes’ viability (Figure 10b). IC_50_ values were found to be 1.7 and 1.8 μg/mL for 24 and 48 h, respectively. Most of the reports suggest that the antileishmanial properties of silver nanoparticles could be attributed to the slow release of silver ions from the nanoparticles’ surface, which destroy the surface of the cell then penetrate the cytoplasm and bind with the target sites. Furthermore, silver nanoparticles can produce reactive oxygen species. It is commonly known that Leishmania is highly sensitive to these oxygen species, and the drug, which could generate ROS, will be an efficient antileishmanial agent.

## 4. Conclusions

The present study demonstrated the synthesis of silver nanoparticles employing an ecofriendly green approach. The aqueous extract of *Eucalyptus camaldulensis* leaves was successfully used as a reducing agent. Optimal factors of AgNO_3_ concentration, the volume ratio of AEECL extract and silver nitrate, and reaction time have been identified to prepare spherical, small size, small size distribution, and stable colloidal nanosilver without adding any capping agents. The green synthesized silver nanoparticles showed significant antileishmanial activity at a low concentration (3.75 µg/mL) against *Leishmania tropica* promastigotes, inhibiting the growth of parasites by 85% after 24 h of incubation. These results could represent a future alternative drug or could be a supportive treatment for Leishmaniasis. In future work, the cytotoxicity of green synthesized colloidal nanosilver on human cell lines such as macrophages will be investigated.

## Figures and Tables

**Table 1:** DPPH radical scavenging ability of CN-Ag and ascorbic acid at different concentrations.

Samples	Concentration (μg/mL)	Scavenging Ability (%)	IC_50_ (μg/mL)
**CN-Ag**	0.5	3.96 ± 0.53	9.46 ± 0.59
	1.25	5.22 ± 0.22	
	2.5	12.88 ± 0.22	
	5	26.33 ± 0.59	
	12.5	66.37 ± 0.91	
**Ascorbic acid**	1	51.90 ± 1.78	0.78 ± 0.15
	2	59.62 ± 0.92	
	3	71.14 ± 0.81	
	4	85.04 ± 0.52	
	5	85.88 ± 1.03	
